# Interleukins and inflammatory markers are useful in predicting the severity of acute pancreatitis

**DOI:** 10.17305/bjbms.2019.4253

**Published:** 2020-02

**Authors:** Davorin Branislav Ćeranić, Milan Zorman, Pavel Skok

**Affiliations:** 1 Department of Gastroenterology, Division of Internal Medicine, University Medical Centre Maribor, Maribor, Slovenia; 2 Faculty of Electrical Engineering and Computer Science, University of Maribor, Maribor, Slovenia; 3 Faculty of Medicine, University of Maribor, Maribor, Slovenia

**Keywords:** Acute pancreatitis, etiology, inflammatory markers, interleukins, scoring systems, Ranson’s criteria, IL6

## Abstract

Acute pancreatitis (AP) is a disease with significant morbidity and mortality. The aim of this study was to evaluate the predictive role of inflammatory markers, particularly interleukins (ILs), in the course of AP and to determine the frequency of etiologic factors of AP. We included patients with AP who were treated at our institution from May 1, 2012 to January 31, 2015. Different laboratory parameters, including ILs, and the severity scoring systems Ranson’s criteria and Bedside Index of Severity in Acute Pancreatitis (BISAP) were analyzed. AP was classified into mild and severe, and independent parameters were compared between these groups. The predictive performance of each parameter was evaluated using receiver operating characteristic (ROC) curves and the area under the ROC curve (AUC). A binomial logistic regression was performed to evaluate Ranson’s criteria and IL6, IL8, and IL10 (at admission and after 48 hours) in the course of AP. Overall, 96 patients were treated, 59 (61.5%) males and 37 (38.5%) females, average age 62.5 ± 16.8 years (range 22–91 years). The best predictor for the severity of AP was IL6, measured 48 hours after admission (AUC = 0.84). Other useful predictors of the severity of AP were lactate dehydrogenase (*p* < 0.001), serum glucose (*p* < 0.006), and difference in the platelet count (*p* < 0.001) between admission and after 48 hours (*p* < 0.001), hemoglobin (*p* < 0.027) and erythrocytes (*p* < 0.029). The major causes of AP were gallstones and alcohol consumption. According to our results, IL6 and Ranson score are important predictors of the severity of AP.

## INTRODUCTION

Acute pancreatitis (AP) is a common acute disease of the gland, associated with various local and distant complications and significant morbidity and mortality [1-3]. The revised Atlanta classification divides AP according to morphological changes (interstitial/edematous AP, acute peripancreatic fluid collection, pancreatic pseudocyst, acute necrotic collection, walled-off necrosis, and necrotizing AP) and severity of the disease (mild, moderately severe, and severe) [4,5].

Mild AP has a very low mortality rate (<1%), whereas the death rate for severe AP (SAP) can be 10–30% [5-7]. AP patients who develop respiratory, cardiovascular, and/or renal failure within the first five days are at increased risk, with a mortality of 30–50% [5,8-11]. Organ failure is usually defined according to the modified Marshall scoring system [11].

The overall success in treating AP has improved in the past decades [4,5,8]. The course of the disease can be assessed by clinical and laboratory indicators and, additionally, the assessment can be completed by scoring systems to predict risks, complications, and treatment outcomes [12,13]. Pro-inflammatory markers (PIMs) and anti-inflammatory markers (AIMs) have also been used as markers of inflammation in AP [14-16]. PIMs include tumor necrosis factor (TNF)-α, interleukin (IL)-1β, IL-2, IL-6, IL-18, chemokines (IL-8), monocyte chemoattractant protein-1, macrophage inflammatory protein-1, growth-regulated oncogene-α, adhesive molecules, platelet-activating factor (PAF), various reactive oxygen and nitrogen compounds [2,3,14-16]. AIMs include IL-10, IL-11, and IL-1 receptor antagonist (IL-1RA) [14,16-18].

Currently, the Ranson’s criteria, Acute Physiology and Chronic Health Evaluation II (APACHE II) system, and Multiple Organ Dysfunction Score (MODS) are the most widely used in clinical practice for predicting the course of AP [4,5,11-13]. In 2008, the Bedside Index of Severity in AP (BISAP) score has been proposed to identify patients at high risk for severe disease early during the course of AP [13]. Because of the technological advances, the prognostic value of imaging evaluation has improved greatly, especially with computed tomography (CT) and Balthazar score. Additionally, the prognosis of these patients improved, due to multidisciplinary treatment approach that follows the current guidelines [8,19-23].

Estimation of pro- and anti-inflammatory cytokine response during AP showed good predictive results in some studies [14-16]. TNF, IL-1, IL-6, IL-8, IL-1β, PAF, leukotrienes, and lipolytic and proteolytic enzymes are important among the pro-inflammatory cytokines [14-17]. On the other hand, anti-inflammatory cytokines are responsible for decreasing the activity of pro-inflammatory cytokines and for reducing the inflammation. IL-4, IL-10, IL-11, IL-13, and IL-1RA are the most important among anti-inflammatory cytokines [14,16-18].

The incidence rate of AP varies in different countries. It depends on the age, gender, and dietary habits of patients, ranging from 10/100,000 inhabitants in England to 70/100,000 in Finland and 80/100,000 in the USA [24-30]. Unfortunately, the etiology of AP remains unexplained in 10% of patients [31].

The aims of this study were to determine the usefulness of inflammatory markers, particularly interleukins, in the prediction of severity of AP.

## MATERIALS AND METHODS

A total of 121 patients, who were treated at the Department of Gastroenterology (Division of Internal Medicine) in the period from May 1, 2012 to January 31, 2015, were included in the study. Diagnosis of AP was confirmed by fulfilling at least 2 of 3 criteria of the revised Atlanta classification (abdominal pain, at least a 3-fold increase in the activity of amylase and lipase, and imaging findings). Patients with known malignant disease and patients who underwent abdominal surgery 30 days prior to admittance were not included in the study.

### Ethics

The study was approved by the National Medical Ethics Committee of the Republic of Slovenia, No. 36/11/09. Written informed consent was obtained from patients who participated in this study.

### Laboratory tests

On admittance and during the treatment, patients underwent the following laboratory tests: white blood cells (WBC), red blood cells (RBC), hematocrit (Ht), hemoglobin (Hb), potassium, sodium, chloride, calcium, C-reactive protein (CRP), liver function tests, amylase, lipase, serum glucose, cholesterol, triglycerides, blood urea nitrogen (BUN), creatinine, lactate dehydrogenase (LDH), iron, ferritin, proteinogram, blood coagulation factors, IL-1, IL-6, IL-8, and IL-10. The determination of ILs was carried out in an international approved laboratory of the Department of Laboratory Diagnostics of the University Clinical Center Maribor with the commercial tests, the chemiluminescence method, and with the analyzer Immulite/Immulite 1000 (Siemens Healthcare Diagnostics Inc., Newark, NJ, USA), according to the manufacturer’s instructions. Biochemistry tests were performed using a spectrophotometric method on the analyzer Dimension Vista System 1500 (Siemens Healthcare Diagnostics Inc., Newark, NJ, USA).

### Imaging methods in AP

Imaging tests (abdominal X-ray at admission, to exclude perforation or ileus, and abdominal ultrasound [US]) were performed within the first 24 h after admission. Abdominal contrast enhanced CT (CECT), endoscopic ultrasound (EUS), and magnetic resonance cholangiopancreatography (MRCP) were performed when the diagnosis was unsure, or if a complication was assumed. Endoscopic retrograde cholangiopancreatography (ERCP) with papillotomy was performed in patients with choledocholithiasis. CECT was repeated in patients with complications such as pseudocysts and pancreatic necrosis.

A multidisciplinary approach was used in all patients (radiologist, gastroenterologist, abdominal surgeon, and anesthesiologist).

### Treatment of AP

Patients received the following treatment: infusion treatment according to cardiac and kidney function; electrolytes replacement with potassium chloride or calcium gluconate; analgesics, tramadol or metamizole, and piritramide (spasmolytic trospium chloride proton pump inhibitor pantoprazole nitrate patch, if there was suspicion of Oddi sphincter spasms); and intravenous broad-spectrum antibiotics, ceftriaxone or ciprofloxacin, and metronidazole or imipenem/cilastatin, in cases of infections and elevated serum inflammation markers (CRP >150 mmol/l, Leukocytes >12.000/ml, procalcitonin >1.3 and positive blood, urine, or sputum culture).

### Assessment of the severity of AP

The severity of AP was assessed in accordance with the scoring systems (Ranson >3, BISAP >3, and MODS >2) as a mild, SAP, or AP with complications. AP without complications was considered as mild AP. However, an AP accompanied by local and/or systemic complications was considered as SAP. Local complications included acute peripancreatic fluid collection, pancreatic pseudocyst, and pancreatic necrosis or abscess. Systemic complications included systemic inflammatory response syndrome (SIRS) and/or single organ failure. SIRS was defined by the presence of at least two out of the four following criteria: body temperature >38°C or <36°C, respiratory rate >20/min or pCO2 >32 mm Hg, heart rate >90/min, WBC >12.000/mm^3^ or <4000/mm^3^, or immature neutrophils >10%. Cultures of blood, urine, stool, sputum, or wound smears were done in cases of extrapancreatic site infections. Mortality was defined as death during hospital treatment or within 30 days after discharge.

### Statistical methods

Data were analyzed using IBM SPSS Statistics for Windows, Version 23.0. (IBM Corp., Armonk, NY). Results were expressed as means and 95% confidence intervals since the results were not distributed equally. The Wilcoxon test for dependent samples was used to compare parameter values received at admission and 48 h after admission (follow-up). The non-parametric Mann–Whitney U-test was used to compare independent parameters in patients with mild and severe courses of the disease. The predictive performance of each parameter was evaluated using receiver operating characteristic (ROC) curves and the area under the ROC curve (AUC). Sensitivity, specificity, positive predictive value (PPV), negative predictive value (NPV), and accuracy were calculated. The value of *p* < 0.05 was considered as statistically significant. Binomial logistic regression was performed to determine the effects of ILs on the severity of the disease.

## RESULTS

One hundred and twenty-one patients were included in this prospective study. Twenty-five patients were lost from the study due to inadequate compliance in patients with alcoholic pancreatitis. We analyzed data of 96 patients. There were 59 (61.5%) male and 37 (38.5%) female patients, with the mean age of 62.5 ± 16.8 years, ranging from 22 to 91 years.

**FIGURE 1:**
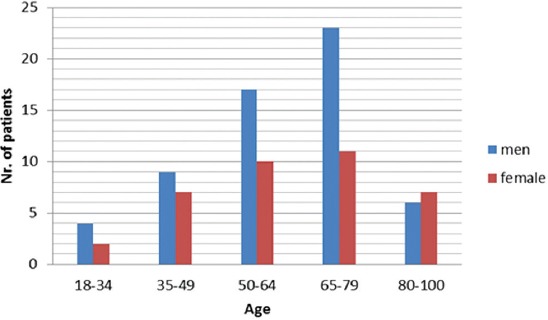
Age and gender distribution of patients with acute pancreatitis. Male patients and those in middle and older age groups were more prevalent in our study population.

Demographic characteristics of patients with AP are presented in Figure 1.

Gallstones were the cause of AP in 54 (56%) patients and excessive alcohol consumption in 26 (27%) patients. Complications secondary to ERCP with endoscopic papillotomy occurred in 5 (5.2%) patients. Drug-induced AP was diagnosed in 2 (2%) patients; in both cases the patients were using azathioprine. The etiology of AP remained unexplained in 9 out of 96 patients (9%).

Recurrent AP was diagnosed in 13 (13.5%) patients: 7 (54%) patients with alcoholic and 6 (46%) patients with biliary etiology. Due to the elevated inflammatory markers within 48 h after the admission, 77% of patients received broad-spectrum antibiotic therapy, most commonly a combination of cephalosporin or quinolone and metronidazole.

The average duration of hospital stay for all patients was 12.0 ± 8.2 days, with a range of 3–75 days. Three male patients (3/93; 3.1%) died due to associated diseases (one patient due to diabetes with complications and two patients due to heart failure). After discharge, 33% of patients (32/96) with gallstones were referred with priority to cholecystectomy (two patients refused surgical treatment).

### Risk stratification of AP

All patients who were included in the study were stratified according to the Ranson’s criteria, with an average score of 2.3 ± 1.53 and a range of 1–7. There were 83% of patients (80/96) with mild AP and 17% of patients (16/96) with SAP. Severity staging, according to the BISAP score, was 0.95/5 ± 0.74 points, ranging from 0 to 3. We assessed the important parameters for the prediction of disease course regarding mild and severe AP (i.e., IL-6, IL-8, IL-10, CRP, serum amylase values on admission and 48 h after admission; LDH and serum glucose at admission). Values for single parameters are presented in Table 1.

**TABLE 1:**
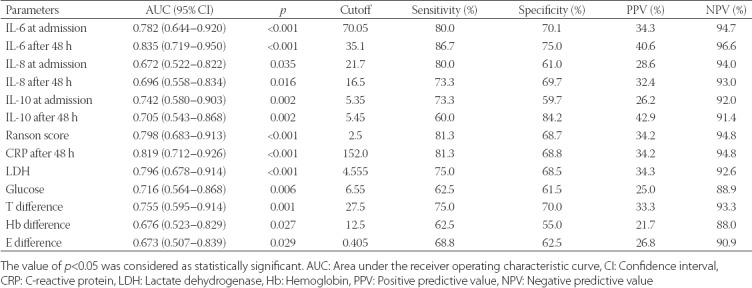
Mann–Whitney U-test: Comparison of independent samples (mild and severe course) by chosen parameters

**FIGURE 2:**
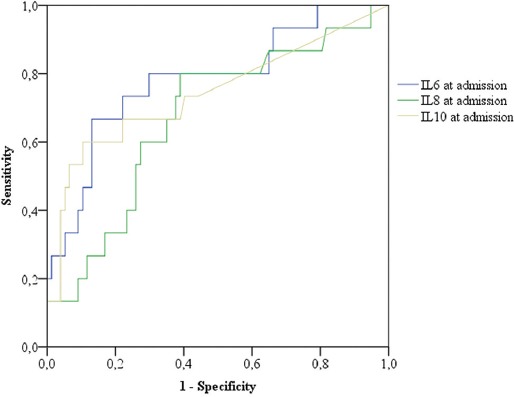
Comparison between predictive values of IL-6, IL-8, and IL-10 at admission in the prediction of severity of AP. The values of IL-6 at admission had the highest predictive value (AUC = 0.782). At a value of IL-6 = 70.05 pg/ml, the sensitivity was 0.80, specificity 0.701, PPV 0.40, and NPV was 0.96 (p < 0.001). IL: Interleukin, AP: Acute pancreatitis, AUC: Area under the receiver operating characteristic curve, PPV: Positive predictive value, NPV: Negative predictive value.

**FIGURE 3:**
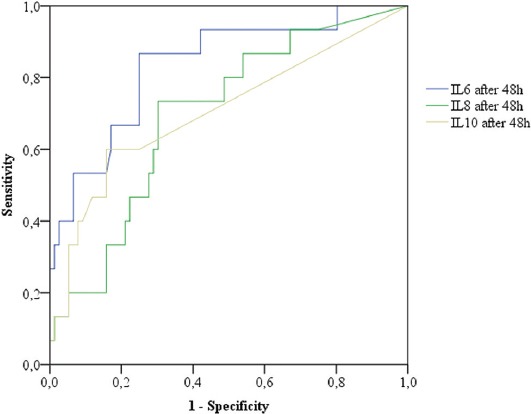
Comparison between predictive values of all interleukins (IL-6, IL-8, and IL-10) 48 h after admission (follow-up) in the prediction of severity of AP. IL-6 had the highest predictive value (AUC = 0.835) in the follow-up. At a value of IL-6 = 36.1 pg/ml the sensitivity was 0.867 and specificity was 0.75. IL: Interleukin, AP: Acute pancreatitis, AUC: Area under the receiver operating characteristic curve.

Our results showed that IL-6 had the greatest predictive value for AP severity at admission and also had the best predictive value for AP severity in the follow-up, compared to IL-8 and IL-10. IL-8 (AUC = 0.70) also demonstrated high predictive value in the follow-up, and IL-10 had the same predictive value for AP severity at admission and in the follow-up [AUC = 0.70] (Figure 2 and 3).

**FIGURE 4:**
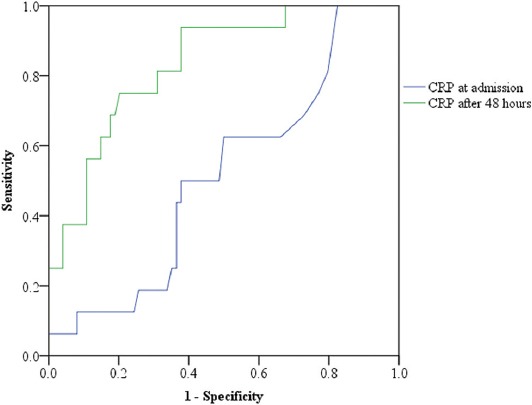
CRP had a greater predictive value for the severity of AP in the follow-up (AUC = 0.82). At a cutoff value of CRP on the third day = 152, the sensitivity was 0.81 and specificity was 0.69. CRP: C-reactive protein, AP: Acute pancreatitis, AUC: Area under the receiver operating characteristic curve.

Comparing the values at admission and 48 h after admission (follow-up), CRP showed the greatest predictive value in the follow-up of AP course (Figure 4).

LDH (*p* < 0.001), serum glucose (*p* < 0.006), difference in the platelet count between admission and 48 h after admission (*p* < 0.001), Hb (*p* < 0.027), and RBC (*p* < 0.029) proved to be statistically significant for predicting the disease course.

Ht (*p* < 0.06), lipase (*p* < 0.18 for the first day and *p* < 0.32 for the third day), and serum amylase (*p* < 0.27 for the first day and *p* < 0.99 for the third day) proved not to be statistically significant for predicting the course of AP.

**FIGURE 5:**
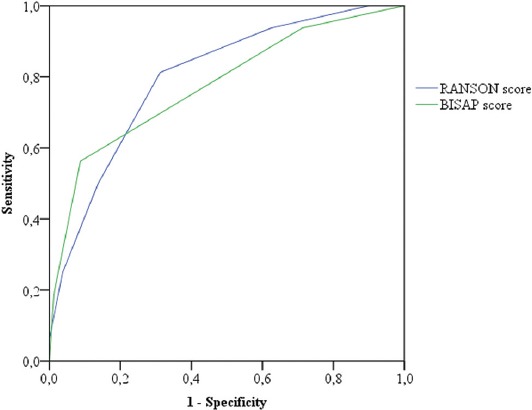
Ranson score had a slightly better predictive value for AP severity compared with the BISAP score. At a cutoff value of Ranson score = 2.5 the sensitivity was 0.813 and specificity was 0.687. At BISAP score = 1.5 the sensitivity was 0.563 and specificity was 0.912. AP: Acute pancreatitis, BISAP: Bedside Index of Severity in Acute Pancreatitis.

Compared with the BISAP scoring system (AUC = 0.78), the Ranson score (AUC = 0.8) had a higher predictive value for the disease course, but this was not statistically significant (Figure 5).

WBC (AUC = 0.70) appeared to have the greatest predictive value for the course of AP among Hb, RBC, Ht, and platelet number. At a WBC value of 11.6, the sensitivity was 0.63 and specificity was 0.71. Among BUN, creatinine, serum calcium, and prothrombin time in the follow-up, BUN had the greatest predictive value for AP course (AUC = 0.70). At a threshold BUN value of 3.75, the sensitivity was 0.73 and specificity was 0.57.

Platelet values at admission and 48 h after admission had the strongest impact on distinguishing between mild AP and SAP (AUC = 0.76; CI = 0.60–0.91). At a cutoff T-value of 27.5, the sensitivity was 75% and specificity was 70%.

### Binomial logistic regression

Binomial logistic regression was performed to determine the effects of 7 variables on the likelihood that participants will have a severe course of the disease, as follows: Ranson score and IL6, IL8, and IL10 measured at admission and 48 h after admission. The linearity of the continuous variables with respect to the logit of the dependent variable was assessed via the Box-Tidwell procedure. A Bonferroni correction was applied using all 15 terms in the model, resulting in statistical significance being accepted when *p* < 0.00333. The logistic regression model was statistically significant, *χ*^2^ (7) = 43.430, *p* < 0.0001. The model explained 64.2% (Nagelkerke R^2^) of the variance in severity of the course of the disease and correctly classified 93.4% of cases. Sensitivity was 98.7%, specificity was 66.7%, PPV was 90.9%, and NPV was 93.8%. Of the 7 predictor variables, only two were statistically significant: Ranson score and IL6 after 48 h (Table 2). A higher Ranson score was associated with an increase in the likelihood of exhibiting severe course of the disease. Similarly, an increasing IL6 value 48 h after admission was associated with an increase in the likelihood of exhibiting severe course of the disease.

**TABLE 2:**
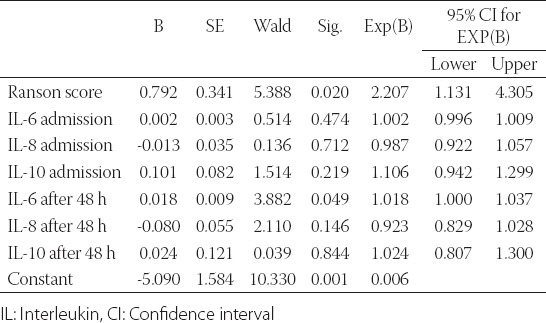
Binomial logistic regression

## DISCUSSION

We confirmed an important role of inflammatory markers, in particular, interleukins, in the prediction of severity in the follow-up of patients with AP. AP is still a disease with significant morbidity and mortality. The incidence of the disease is very different in different countries and is associated with dietary and other habits of patients. With this prospective study in a tertiary institution, we wanted to determine the etiology of AP and whether modern imaging methods and laboratory findings, including interleukins, can predict the course of the disease accurately and delineate between mild and severe AP forms.

Gallstones and alcohol were the most common causes of AP, which is in accordance with the previous reports [1,24,25,28,29]. In recent years, the number of younger patients with AP increased in our, as well as in other regions, which may be correlated with the increased alcohol consumption among younger population [24-26,28]. The average length of stay (12 days) and mortality rate (3%) in Slovenia are comparable with other developed countries, as well as the proportion and type of complications (e.g, pancreatic necrosis and pseudocyst) [1,5,22,28].

LDH, serum glucose, difference in the platelet count between admission and 48 h after admission, Hb, and RBC proved to be statistically significant in predicting the disease course [3,14,16].

Several laboratory findings and prediction models have been used to estimate the course of AP. Interleukins – an unstructured group of proteins secreted by numerous cells in the body, such as monocytes, macrophages, endothelium, and fibroblasts, are produced as a response to a proinflammatory stimulus, and are useful in predicting the course of AP [2,3,14-17]. ILs enable cell growth, differentiation, circulation, and take a part in the inflammatory process and immune response. They also promote healing and recovery. Here, we confirm the positive role of IL-6 in the prediction of SAP at admission as well as in the follow-up 48 hours after admission [15-17]. Early diagnosis and differentiation between mild and SAP using simple and quick assessment is necessary in everyday clinical practice [4,5,7,11,12]. Pancreatic enzymes are useful only for diagnosis and not for prognosis of AP. Two studies confirmed that the levels of IL-6 as well as IL-8 and IL-10 are higher in patients with SAP [16,17]. On the other hand, because of the fast decrease in serum, IL-6 is not a good marker for longer monitoring of the disease.

Gunjaca et al. [16] studied pro-inflammatory and anti-inflammatory processes in AP. Pro-inflammatory cytokines were significant for the pathogenesis of SAP, and IL-6 was recognized as a key mediator in acute phase protein synthesis. Using a logistic regression analysis, they showed even a better independent prognostic value for IL-10 (better than for IL-6) [16].

In the present study, CRP was a good predictive factor after 48–72 h of admission. It is well-known that IL-6 increases earlier than IL-8 during the inflammatory process and induces the synthesis of CRP (and other acute-phase proteins). CRP had the best predictive value for the severity of AP after admission, with the AUC of 0.82. At a value of CRP = 152, the sensitivity was 0.81, specificity 0.69, PPV 0.34, and NPV was 0.95. Digalakis et al. [32] also studied CRP, IL-8, and TNF-α as predictors of the severity of AP. CRP originates in the liver and is induced by the release of IL-1 and IL-6. They showed higher serum levels of IL-8 in patients with SAP compared with patients with mild or moderate AP, and established the highest sensitivity and diagnostic accuracy in predicting severity on the second day of hospitalization [32]. Başak et al. [33] used Ranson score and CRP in predicting the severity of AP. They demonstrated that the concomitant use of CRP and Ranson score could distinguish SAP from mild AP, with the sensitivity, specificity, and accuracy of CRP of 82.9%, 80.9%, and 81.1%, respectively [33]. Vasseur et al. [18] analyzed the role of IL-22 in AP course and showed a high increase during the early phase of AP.

Gunjaca et al. [16] and Fisic et al. [17] indicated that IL-6 and IL-10 are useful markers of the AP severity; however, their use in routine clinical practice is limited because of their high costs. The analysis of ILs is not possible in all laboratories. The other good predictors in our analysis are available in all laboratories and are easy to evaluate, such as LDH, serum glucose, BUN, difference in the values of platelets between the first and the third day, Hb, and RBC [34]. However, their role and accuracy should be confirmed in larger randomized studies.

Lack of data on the most difficult patients treated in intensive care units is the limitation of this study, since only 17% of patients had SAP, indicating less severe disease in this group compared with the previous studies.

It is necessary to provide a critical evaluation of treatment results, as well as material and non-material factors affecting the treatment. In addition, restrictions applied in certain environments must also be taken into consideration. When treating patients who are most at risk, it is necessary to provide a multidisciplinary approach in making optimal clinical decisions [5,8,10,22,28]. This is the only approach that enables a modern and successful treatment of patients suffering from AP. The authors of this study also share the opinion that the outcome of treatment of mild or severe AP undoubtedly influences the implementation of modern recommendations for the management of this disease [23]. IL-6 is a useful marker for the AP course; however, its use in clinical practice is limited because of its high costs.

CRP, LDH, serum glucose, BUN, difference in the platelet count between admission and 48 h after admission, Hb, and RBC were all good predictive factors for AP severity in our study; however, their role and accuracy should be confirmed in larger randomized studies.

Despite new inflammatory markers introduced and numerous studies determining laboratory values and models for predicting disease severity in patients with AP, it seems that the Ranson’s criteria remain to be useful in the validation of AP in everyday clinical practice, which strongly supports their use for that purpose.

Gallstones and alcohol were the leading causes of AP in our population.

## CONCLUSION

Inflammatory markers, in particular interleukins, are important tools in the prediction of severity and in the follow-up of patients with AP. Unfortunately, low availability and high costs are limiting their use in everyday clinical practice. However, Ranson score, CRP, WBC, platelets, and BUN are simple and available markers for routine clinical work.

The multidisciplinary approach and implementation of modern recommendations in clinical practice will enable successful treatment and lower mortality in patients with AP.

## DECLARATION OF INTERESTS

The authors declare no conflict of interests.
